# High Drug-Loading Nanomedicines for Tumor Chemo–Photo Combination Therapy: Advances and Perspectives

**DOI:** 10.3390/pharmaceutics14081735

**Published:** 2022-08-19

**Authors:** Ya Wang, Yujie Zhang, Xiaojiang Zhang, Zhe Zhang, Junjun She, Daocheng Wu, Wei Gao

**Affiliations:** 1 Center for Gut Microbiome Research, Med-X Institute, The First Affiliated Hospital of Xi’an Jiaotong University, 277 West Yanta Road, Xi’an 710061, China; 2 Department of General Surgery, The First Affiliated Hospital of Xi’an Jiaotong University, 277 West Yanta Road, Xi’an 710061, China; 3 Key Laboratory of Biomedical Information Engineering of the Ministry of Education, School of Life Science and Technology, Xi’an Jiaotong University, Xi’an 710049, China; 4 Department of Anesthesiology & Center for Brain Science & Center for Translational Medicine, The First Affiliated Hospital of Xi’an Jiaotong University, Xi’an 710061, China

**Keywords:** high drug-loading nanomedicines, chemotherapy, phototherapy, combination therapy

## Abstract

The combination of phototherapy and chemotherapy (chemo–photo combination therapy) is an excellent attempt for tumor treatment. The key requirement of this technology is the high drug-loading nanomedicines, which can load either chemotherapy drugs or phototherapy agents at the same nanomedicines and simultaneously deliver them to tumors, and play a multimode therapeutic role for tumor treatment. These nanomedicines have high drug-loading efficiency (>30%) and good tumor combination therapeutic effect with important clinical application potential. Although there are many reports of high drug-loading nanomedicines for tumor therapy at present, systematic analyses on those nanomedicines remain lacking and a comprehensive review is urgently needed. In this review, we systematically analyze the current status of developed high drug-loading nanomedicines for tumor chemo–photo combination therapy and summarize their types, methods, drug-loading properties, in vitro and in vivo applications. The shortcomings of the existing high drug-loading nanomedicines for tumor chemo–photo combination therapy and the possible prospective development direction are also discussed. We hope to attract more attention for researchers in different academic fields, provide new insights into the research of tumor therapy and drug delivery system and develop these nanomedicines as the useful tool for tumor chemo–photo combination therapy in the future.

## 1. Introduction

Tumors are of the most serious diseases affecting human health because of their high incidence rate and mortality worldwide [1]. Traditional surgical resection, radiotherapy, and chemotherapy are the main methods of tumor treatment, but each method has its limitations. Surgical resection and radiotherapy are effective for local nonmetastatic tumors but do not work for metastatic tumors and tumors growing at critical sites. Chemotherapy is an effective systemic treatment for primary and metastatic tumors. It uses chemical agents to kill and inhibit the rapid proliferation and metastasis of tumor cells in a long duration. However, the application of chemotherapeutic drugs is limited by their poor targeting and dose-limiting toxicity, problems of high-toxicity side effects, tumor–drug resistance, incomplete tumor killing, and high tumor recurrence rate [2,3]. Therefore, new therapy methods that can reduce systemic toxicity and improve the efficiency of comprehensive treatment are urgent to seek.

With the development of tumor-therapy technology, the emerging gene therapy, immunotherapy, and physical therapy (including magnetic therapy, hyperthermia, phototherapy, and ultrasonic therapy) are gradually being applied to clinical tumor treatment [4]. Among them, light-induced phototherapy is a promising “green” physical therapy [5,6]. This method uses near-infrared (NIR) light with tissue-penetration ability to irradiate tissue containing the concentrated phototherapeutic agents. It can selectively kill the tumor cells without inflicting too much damage to normal tissue. Recently, phototherapy has attracted extensive attention owing to its advantages of non-invasiveness, low toxicity side effects, and efficient and accurate treatment [7]. Photothermal therapy (PTT) and photodynamic therapy (PDT) are two of the most important phototherapies for tumor treatment. PTT converts light energy into heat energy through photothermal agents, and the resulting local high temperature leads to the rapid ablation and death of tumor cells [8]. Different from PTT, PDT uses singlet oxygen or reactive oxygen species (ROS) generated from photosensitizer (PS) molecules under light exposure to kill tumor cells [9]. Phototherapy has the advantages of minimal invasiveness, simple operation, quick onset, high curative effect, and low-toxicity side effects. However, light cannot fully cover the focus and has limited the penetration depth in the tumor tissue and short treatment duration, resulting in the survival of the tumor cells at the edge or deep in the tumor and tumor recrudescence [5,10]. The combination of phototherapy and chemotherapy (chemo–photo combination therapy) is a good attempt for tumor treatment, which can integrate the advantages of each treatment and overcome the defects of the two methods, improve the tumor treatment effect, and reduce the toxic side effects. Therefore, chemo–photo combination therapy, as an important combined tumor-therapy technology, has great potential in clinical applications.

The key requirements of chemo–photo combination therapy are the delivery of chemotherapeutic agents and phototherapeutic agents to the tumor site at the same time. With the development of nanotechnology, nanomedicines provide more possibilities for chemo–photo combination therapy, which can simultaneously load chemotherapy drugs, PSs, and photothermal agents, and deliver them to tumors to play a multimode therapeutic role. So far, there have been several related reviews published, reporting either organic or inorganic nanomaterials for chemo–photo combination therapy [11,12]. Li et al. reviewed the research progress of advanced nanomaterials in chemo–photothermal combination therapy in recent years, mainly including metal based and carbon based nanomaterials, with emphasis on organic nanomaterials [13]. Khafaji et al. reviewed most of the new strategies of inorganic nanostructures for cancer chemo–photothermal combination therapy and cancer imaging, and discussed the current challenges and future prospects in this field [14]. Denkova et al. reviewed the basic requirements for the design of nanocarrier systems combining PDT and PTT with chemotherapy or radiotherapy [15]. Nevertheless, the ideal nanomedicines for chemo–photo combination therapy should be loaded with as many chemotherapeutic agents and PSs as possible. The optimum ratio between the carrier and therapeutic agent should be determined to fully exploit the best combination therapy effect. The combined treatment system of chemo–photo combination therapy with a drug-load rate of more than 30% is called high drug-loading nanomedicines, which include different nanosized formulations such as nanoparticles (NPs), nanodots, nanolayer, nanorods, and nanocomposite. For example, Zhang et al. prepared doxorubicin (DOX)-loaded spherical polydopamine (PDA)/mesoporous calcium phosphate Janus nanoparticles (PDA/mCaP H-JNPs), selectively functionalized its surface with indocyanine green (ICG) and methoxy poly(ethylene glycol) (PEG) mercaptan as the nanomedicines for photoacoustic image-guided chemotherapy–photothermal collaborative tumor therapy [16]. The synthesized NPs have excellent drug-loading efficiency (92%), high photothermal conversion efficiency (45.3%), and NIR and pH dual-response drug-release performance. In vivo antitumor experiments show that the tumor-inhibition rate (TIR) of combined chemotherapy and PTT mediated by PEG–ICG–PDA/mCaP H-JNPs is 96.2%. Liu et al. developed functionalized MoS_2_ nanosheets as a multifunctional nanomedicine for tumor chemo–photo combination therapy [17]. By chemically stripping MoS_2_ nanosheets and functionalizing them with lipoic acid modified PEG (LA-PEG) through thiol reaction, chemotherapy drugs such as DOX, 7-ethyl-10-hydroxy-camptothecin (SN38), and photodynamic agent chlorine E6 (Ce6) can be loaded effectively. Under their test conditions, the loading capacities of DOX, Ce6, and SN38 to MoS_2_-PEG were improved as the drug concentration increased, with the highest drug-load ratios (weight ratio of drug to MoS_2_) being 239% of DOX, 39% of Ce6, and 118% of SN38. In vivo treatment experiments have shown that MoS_2_-PEG/DOX can achieve significant combination anticancer effects through intratomatous and intravenous injection. Feng et al. prepared PEGylated bismuth tungstate nanosheets loaded with DOX (Bi_2_WO_6_-DOX-PEG NSs) for the combined chemotherapy/PDT/PTT of tumors [18]. The Bi_2_WO_6_-DOX-PEG NSs not only possess excellent drug-loading efficiency (81.3%), but also can efficiently produce ROS and high hyperthermia effect (36.5% of heat transfer coefficient). In vitro and in vivo experiments showed that Bi_2_WO_6_-DOX-PEG NSs had negligible systemic toxicity and good tumor ablation ability. Considerable high drug-loading nanomedicines for tumor chemo–photo combination therapy have been developed, showing good combination antitumor efficiency and low-toxicity side effects. However, systematic analyses on this kind of nanomedicines remain lacking. Accordingly, a comprehensive review of high drug-loading nanomedicines for tumor chemo–photo combination therapy is urgently needed.

**Figure 1:**
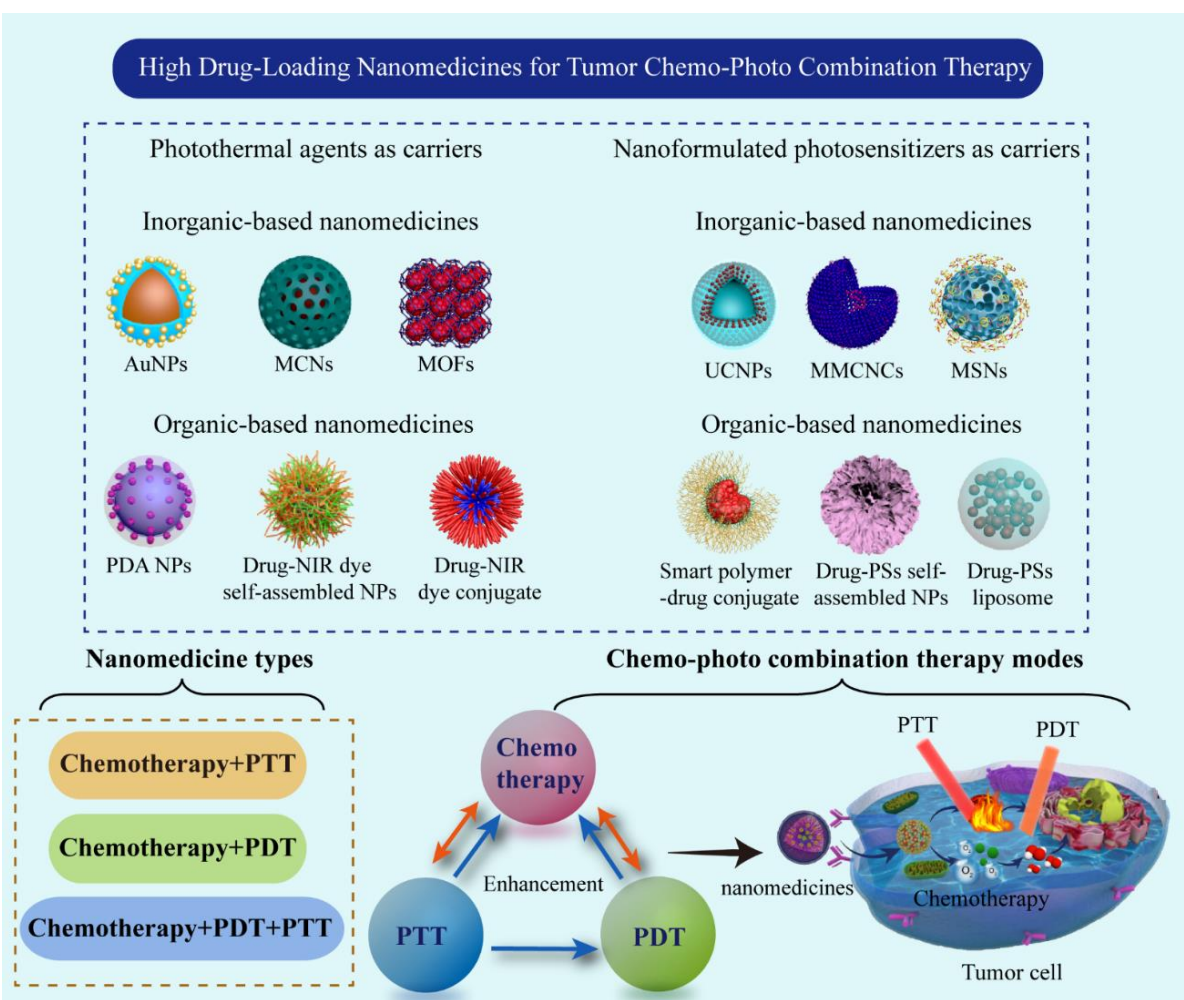
Schematic illustration of this review. AuNPs, gold nanoparticles; MCNs, mesoporous carbon nanospheres; MOFs, metal–organic frameworks; UCNPs, upconversion nanoparticles; MMCNCs, mesoporous magnetic colloidal nanocrystal clusters; MSNs, mesoporous silica nanoparticles.

Considerable reviews have been reported on the combination of chemotherapy and other monotherapies using nanotechnology in recent years. The high drug-loading nanomedicines for tumor treatment are reported by a few scholars with a focus on their construction strategies, preparation methods, and material compositions [19,20]. However, little literature reports on chemo–photo combination tumor therapy by using the high drug-loading nanomedicines, which can be one of the most important research fields in tumor treatment. As shown in Figure 1, the present review systematically analyzes the current status of developed high drug-loading nanomedicines for tumor chemo–photo combination therapy and summarizes their types, methods, drug-load properties, and in vitro and in vivo applications. The shortcomings of the existing high drug-loading nanomedicines and the possible prospective development direction in the future are also discussed. As for research significance, chemo–photo combination tumor therapy and high drug-loading nanomedicines are important directions and trends to improve tumor treatment efficacy in the future, but they have received insufficient attention, in particular the latter. This review can attract more research attention for researchers in different academic fields and provide new insights into the development of chemo–photo combination therapy in the future.

## 2. Phototherapeutic Agents as Carriers for Fabricating High Drug-Loading Nanomedicines

On the premise of a stable system, the lesser amount of carrier material and the higher drug-loading ratio are pursued to prepare the high drug-loading nanomedicines. For tumor chemo–photo combination therapy, chemotherapeutic drugs and phototherapeutic agents (photothermal agents and PSs) are equally important in the nanomedicines, and their proportion determines the final therapeutic effect on tumor to a certain extent. However, the construction of nanomedicines to realize the high loading of chemotherapeutic drugs and ensure the effective dose of phototherapeutic agents in the same nanomedicine is a challenge. After long-term exploration, researchers have found that using a phototherapeutic agent as a carrier to load chemotherapeutic drugs is an effective method of realizing high drug-loading for chemo–photo combination therapy. Now, many kinds of photothermal materials or PSs have been used as carrier materials to construct high drug-loading nanomedicines for tumor combination therapy of chemo-PDT, chemo-PTT, and chemo-PDT-PTT.

### 2.1. Photothermal Agents as Carriers for Fabricating High Drug-Loading Nanomedicines 

Photothermal agents absorb the energy of NIR light and convert it into heat. Consequently, local tumors are induced to generate high temperatures, leading to their thermal ablation or thermal damage, thereby killing tumor cells with less harm to healthy tissues. At present, many photothermal agents, especially nanomaterials with strong absorption capacity and high photothermal-conversion efficiency in the NIR, have been widely studied and explored with some already undergoing clinical trials [6]. These photothermal agents can be primarily divided into inorganic and organic materials. Inorganic materials also include noble-metal nanomaterials, transition-metal chalcogenides and oxides, and carbon-based materials. Numerous studies have shown that nanomedicines prepared using a photothermal agent as a nanocarrier loaded with chemotherapy drugs have high drug-loading and high combination efficiency of chemotherapy-PTT [5]. In this section, chemo-PTT combination therapy with common photothermal agents used as carrier materials are summarized in Table 1.

#### 2.1.1. Inorganic Materials

**Figure 2:**
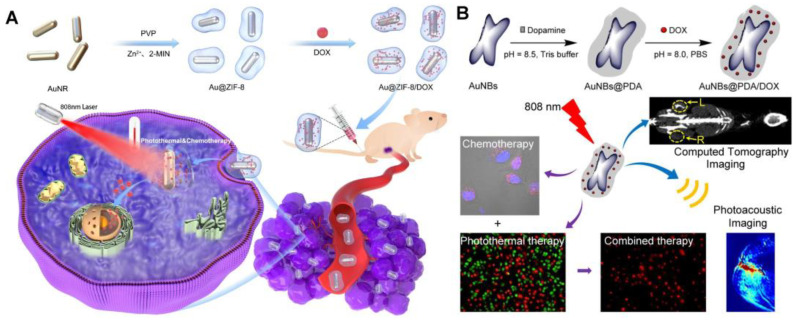
Preparation of high drug-loading nanomedicines from gold-based nanomaterials for combined chemotherapy-photothermal treatment of tumors. (**A**) The schematic illustration of the synthetic procedure of Au@ZIF-8/DOX nanocomplexes for the chemo–photothermal synergistic tumor therapy in vivo [60]. (**B**) The diagram of the AuNBs@PDA/DOX nanomedicines preparation and the nanomedicines used for PA/CT imaging-guided chemo–photothermal therapy of tumor [23]. Reprinted with permission from Ref. [23]. Copyright 2021, Elsevier.

Noble-metal-based materials (Au, Ag, and Pb) are extensively used in tumor imaging and PTT due to their strong localized surface plasmon resonance (LSPR) and enhanced photothermal-conversion efficiency in the NIR-light region [48]. Among them, gold (Au)-based nanomaterials have excellent performance for clinical translation owing to their high X-ray attenuation coefficient, excellent biocompatibility, and bio-inertness. Accordingly, the preparation of high drug-loading nanomedicines with Au-based materials such as gold NPs (Au NPs) [49,50,51], Au nanorods (Au NRs) [52,53], and Au nanocages (Au NCs) [54,55], Au nanoflowers (Au NFs) [56,57], and Au nanoclusters (Au NCSs) [58,59] are attracting extensive attention for chemo–photo combination tumor therapy. Huang et al. used crystalline zeolitic imidazolate framework-8 (ZIF-8) to grow on Au NRs and prepare biocompatible and biodegradable metal–organic frameworks (MOFs), i.e., Au@ZIF-8 [60] (Figure 2A). Due to the large surface area and guest-matching pore size of ZIF-8, DOX is successfully loaded into Au@ZIF-8 with a high drug load efficiency of ~37%. Au@ZIF-8 shows a high photothermal effect upon NIR irradiation, and the ZIF-8 layer quickly degrades with laser irradiation, resulting in on-demand drug release at the tumor site. Furthermore, in vivo therapeutic results confirm that the combination of chemo–photothermal with Au@ZIF-8/DOX + NIR achieves much higher treatment efficacy than that of PTT with Au@ZIF-8 + NIR or chemotherapy with DOX only, even resulting in complete tumor elimination without obvious adverse effect. Xu et al. fabricated PDA-coated Au nanobone (NB) nanocomplexes (AuNBs@PDA) through the in situ polymerization of dopamine on the surface of Au NBs. The DOX is loaded through π-π stacking and hydrogen-binding interactions with PDA [23] (Figure 2B). The AuNBs@PDA nanocomplexes exhibit higher photothermal-conversion efficiency (75.48%) than Au NBs alone, which is beneficial to photoacoustic imaging and PTT.

Moreover, the load efficiency (LE) of DOX by AuNBs@PDA nanomedicines could reach up to about 70%, showing significant cytotoxicity and antitumor effect. He et al. prepared metal@MOFs (Au@Cu_3_(BTC)_2_NPs) with a core–shell structure by assembling Cu_3_(BTC)_2_ on Au NPs with 4-mercaptobenzoic acid (4-MBA) as bridging molecule [61]. The Cu_3_(BTC)_2_ shell can provide sites for aptamer functionalization and drug loading. The Au@Cu_3_(BTC)_2_NPs exhibit high drug-loading efficiency (57%) and good photothermal-conversion efficiency.

**Figure 3:**
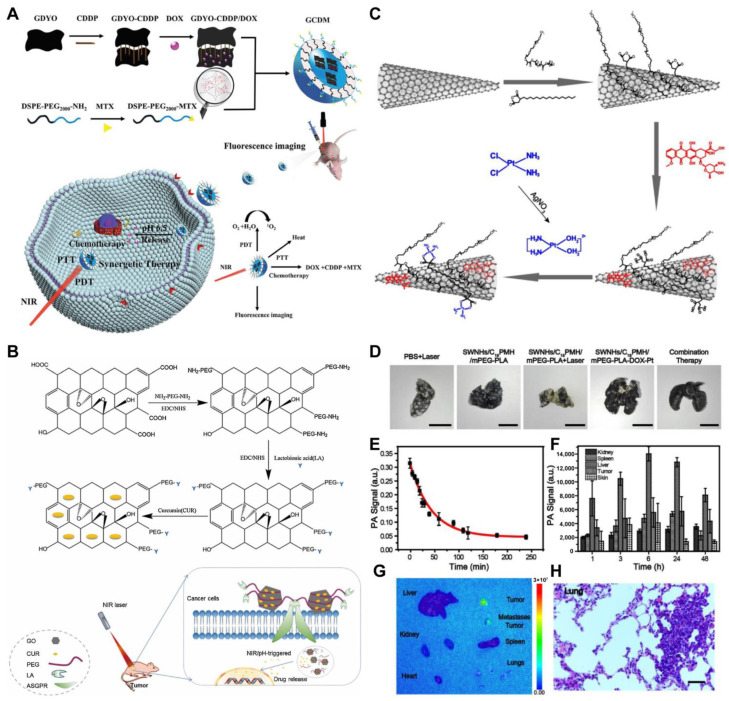
Preparation of high drug-loading nanomedicines from carbon nanomaterials for combined chemotherapy-photothermal treatment of tumors. (**A**) Schematic illustration for the preparation of GCDM [33]. (**B**) The preparation of GO-PEG/LA-CUR nanocomposites [64]. (**C**) Schematic illustration of the preparation of dual drug-loaded SWNHs. (**D**) India ink staining of lungs from different treatment groups. Scale bar is 1 cm. (**E**) Blood circulation time and (**F**) bio-distribution of SWNHs/C18PMH/mPEG-PLA-DOX-Pt evaluated by PAI. (**G**) Ex vivo fluorescence images of major organs and tumors 24 h post injection of SWNHs/C18PMH/mPEG-PLA-DOX-Pt. (**H**) HE staining of lung tissues after fluorescence imaging. The metastatic nodules can be clearly observed. Scale bar is 100 μm [37]. Reprinted with permission from Ref. [33]. Copyright 2021, John Wiley & Sons, Inc. Reprinted with permission from Ref. [64]. Copyright 2020, Elsevier.

Carbon nanomaterials primarily include the zero-dimensional (0D) fullerenes, the one-dimensional (1D) carbon nanotubes (CNTs), and the two-dimensional (2D) graphene [6]. Given the high NIR absorption coefficient (6.2 × 10^6^ M^−1^ cm^−1^) and ultra-high specific surface area of CNTs and graphene, the preparation of high drug-loading nanomedicines based on these materials for tumor chemo–photothermal combination therapy is attracting considerable attention [62,63]. However, CNTs and graphene without appropriate surface functionalization cause toxicity and side effects because of the poor dispersibility and stability of carbon-based materials in physiological solutions. Accordingly, researchers have developed various surface-functionalized CNTs or graphene nanocarriers to load chemotherapeutic drugs and prepared high drug-loading nanomedicines for tumor chemo–photothermal combination therapy. Xing et al. synthesized a three-dimensional framework with good stability and large specific surface area through the amide reaction of graphdiyne oxide (GDYO) and cisplatin (CDDP) and then loaded DOX by π-π stacking [33]. Self-assembly is then performed with the targeting group DSPE-PEG2000-methotrexate (MTX) and GDYO-CDDP to obtain multifunctional nanomedicines of GDYO-CDDP/DOX@DSPE-PEG-MTX (GCDM) for diagnosis and targeted cancer chemo–photothermal combination therapy (Figure 3A). In this nanomedicines, three traditional anticancer drugs (CDDP, DOX, and MTX) play new roles and can reduce multidrug resistance through combined antitumor effects. Although only 40.3% of DOX is given, the load efficiency of the three chemotherapy drugs is actually higher. Wang et al. modified the surface of graphene oxide (GO) nanosheets with PEG derivatives and lactobionic acid (LA), and then loaded curcumin (CUR) on graphene nanosheets by π-π stacking to prepare GO-PEG/LA-CUR composite nanomedicines with a drug-loading efficiency of 56.8% [64] (Figure 3B). In vivo experiments show that tumor growth is significantly inhibited in subcutaneous hepatocellular carcinoma tumor-bearing mice treated with GO-PEG/LA-CUR after NIR irradiation with a tumor inhibition rate of 86%, demonstrating the higher efficacy of the chemo-PTT combination therapy. Yang et al. designed a highly potent chemo–photothermal theragnostic system by sequentially loading DOX and CDDP onto dual polymer-modified single-wall carbon nanohorns (SWNHs) [37]. SWNHs are first modified simultaneously with poly(maleic anhydride-alt-1-octadecene) (C18PMH) and methoxyPEG-b-poly-D,L-lactide (mPEG-PLA) through hydrophobic–hydrophobic interactions and π-π stacking (Figure 3C). Among various carbonaceous materials, SWNHs are advantageous for drug loading and have strong potential for chemo–photothermal combination therapy owing to their unique structure with a horn shape. This closed-tip nanotube possesses large inner voids and can be filled with suitable drugs ranging from small molecules to proteins. In this nanomedicines, the drug-loading efficiency of DOX and CDDP is 44% and 66%, respectively. In vivo treatment results show that SWNHs/C18PMH/MPEG-PLA-DOX-Pt-mediated chemo–photothermal combination therapy with the guidance of photoacoustic imaging completely eliminates the primary breast tumor and inhibits its lung metastasis (Figure 3D–H).

A wide variety of transition-metal compound nanomaterials have opened up a new regime in PTT owing to their excellent NIR absorption and efficient heat-generation abilities. These nanomaterials primarily contain transition-metal chalcogenides (e.g., Cu_x_S_y_ [27,65,66], Cu_2−x_Se [67], CoS [29], MoS_2_ [68], WS_2_ [69], FeSe_2_ [70], FeS [71], and TiS_2_ [72]) and oxides (e.g., MoO_x_ [73], W_x_O_y_ [74], and Ti_8_O_15_ [75]) with different structures of nanosheets, nanodots, and nanoparticles. Among them, copper chalcogenide semiconductors characterized by low cost and toxicity are new kinds of promising photothermal agents. Wu et al. synthesized hollow copper sulfide NPs (HCuSNP) loaded with DOX and a photothermal agent (ICG). They coated the surface of B16 membrane to prepare HCuSNP-ICG-DOX@B16F10 (ID-HCuSNP@B16F10) nanomedicines, which serve as a biomimetic platform for in vivo chemo–photothermal combination therapy of tumors [76]. The hollow mesoporous structure of HCuSNPs makes them an ideal drug carrier. The loading efficiency of ICG and DOX can reach 98% and 85%, respectively. ID-HCuSNP@B16F10 exhibits an excellent photothermal effect in melanoma animal models and achieves a high tumor-ablation rate. As an emerging two-dimensional nanomaterial, MXenes have a large specific surface area and various surface groups, which enable them to have high drug-loading efficiency and the possibility of surface functionalization. Some recent reports have also confirmed the high photothermal-conversion performance of MXenes in NIR PTT. Li et al. developed multifunctional Ti_2_N MXene-based nanomedicines (Ti_2_N@oSi) for chemo–photothermal combination anticancer therapy [77]. Ti_2_N nanosheets are stabilized with soybean phospholipids and loaded with DOX, followed by coating with biodegradable silica shells to prevent drug leakage, and finally loading with the second drug (CDDP) and the target agent (bombesin) in the outer layer. The unique structure of Ti_2_N nanosheets endows the drug carriers with an ultrahigh drug-loading efficiency of 88.8% and an excellent NIR photothermal-conversion efficiency of 41.6% for chemo–photothermal combination therapy. Although inorganic nanomaterials have been developed for the PTT treatment of tumors, most of the inorganic photothermal agents currently used are nonbiodegradable and have potential long-term toxicity, hindering their further application in clinical tumor therapy.

#### 2.1.2. Organic Materials

Organic photothermal agents have excellent biodegradability and biocompatibility. Compared with inorganic photothermal agents, organic agents have fewer safety problems. Thus, the development and application of organic photothermal agents have also been extensively studied for many years.

**Figure 4:**
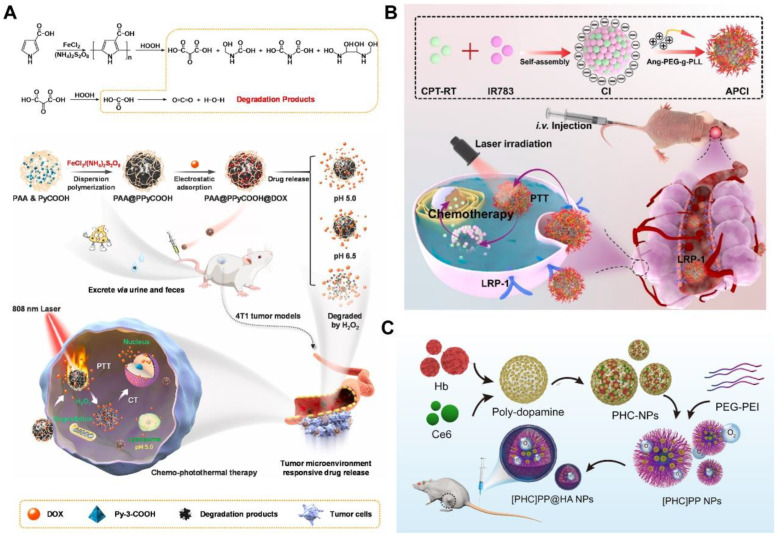
Preparation of high drug-loading nanomedicines from organic nanomaterials for combined chemotherapy-photothermal treatment of tumors. (**A**) Schematic illustration of the preparation of PAA@PPyCOOH@DOX based on degradable conductive polymer PPyCOOH for chemo–photothermal therapy [44]. (**B**) Schematic illustration of preparation of Ang-PEG-g-PLL@CPT-RT@IR783 (APCI) [40]. (**C**) Schematic illustration of the design and prepared lollipop-like nanoparticles assembled with gossypol, doxorubicin, and polydopamine via π-π stacking [78]. Reprinted with permission from Refs. [40,44]. Copyright 2021, Elsevier. Reprinted with permission from Ref. [78]. Copyright 2018, John Wiley & Sons, Inc.

Common organic photothermal agents include NIR-absorbing dye containing nanocomplexes, NIR-absorbing conjugated polymers, and melanin-based photothermal materials. Small-molecule organic dyes such as ICG, IR780, and IR808 are difficult to be loaded with drugs as nanomedicines owing to some of their physicochemical characteristics, such as concentration-dependent aggregation and poor aqueous stability. Accordingly, other carrier materials are often needed. Lu et al. designed two-component NPs by π-π stacking and hydrophobic interactions between amino acid-conjugated camptothecin (CPT-RT) and canine dyes (IR-783) [39]. Then, the positively charged Angiopep-2-modified PEGlyated poly-l-lysine (Ang-PEG-g-PLL) is coated onto the negatively charged two-component NPs by electrostatic interaction to form the three-component nanomedicines Ang-PEG-g-PLL@CPT-RT@IR783 (APCI) for synergistic chemo–photothermal anti-glioma therapy (Figure 4B). The drug-loading efficiency of APCI reaches about 62%. In vivo results reveal that the nanomedicines mediated by chemo–photothermal combination therapy achieve a better therapeutic effect, longer survival time, and minimal toxic side effects in orthotopic glioblastoma tumor-bearing nude mice.

Compared with NIR dyes, conductive polymers such as polypyrrole and polyaniline have better photothermal stability, are not easy to photobleach and have a longer circulation time in vivo [43]. Thus, as a photothermal agent, the conductive polymer is also an ideal drug-delivery platform. Liu et al. fabricated poly(acrylic acid) (PAA)-stabilized poly(pyrrole-3-COOH) NPs (PAA@PPyCOOH NPs) as nanomedicines loaded with DOX for chemo–photothermal combination therapy [44] (Figure 4A). The PAA@PPyCOOH NPs are found to be ideal nanomedicines with good dispersity, excellent biocompatibility, high drug-loading efficiency (43.3%), and photothermal-conversion efficiency (56%). In vivo experiments demonstrate that the PAA@PPyCOOH@DOX nanomedicines can be specifically degraded by excess H_2_O_2_ in the tumor, and the tumor growth of 4T1 breast-cancer model is drastically inhibited by chemo-PTT.

PDA is a kind of melanin-like polymer that has been developed for photothermal agents and nanocarriers because of its excellent biocompatibility, biodegradability, simple preparation conditions, and high photothermal-conversion efficiency. PDA has many functional groups such as benzene ring, catechol group, and quinone group, enabling it to load a large number of antitumor drugs through π-π stacking or hydrogen bonding. In our previous work, we have developed lollipop-like dual-drug-loaded NPs (DOX–PDA–gossypol NPs) based on the self-assembly of gossypol, DOX, and PDA through π-π stacking [78] (Figure 4C). The DOX–PDA–gossypol NPs have a high drug-loading efficiency of 91%. In vivo antitumor experiments show that the TIRs of DOX-PDA-gossypol NPs are more than 90% at both low doses, which is beneficial for widening the drug therapeutic window. In another study, we used PDA as a carrier for loaded hemoglobin and Ce6 to construct small NPs (PHC NPs; about 10 nm) for combined PTT/PDT [79]. The PHC NPs were encapsulated into the micelles formed by aldehyde-modified PEG and polyethyleneimine through benzoic-imine bonds, and the surface was modified with hyaluronic acid to form larger-size composite NPs ([PHC]PP@HA NPs; about 140 nm). The photothermal-conversion efficiency of [PHC]PP@HA NPs is 47.09%, and the load contents of Ce6 and Hb in PHC NPs were 27.3% and 54.8%, respectively. In vivo PTT/PDT experiments show that the TIR in mice is close to 100% within 30 days, and the tumor-recurrence rate is only 8.3% within 60 days. 

In addition to the common nanomaterials mentioned above, researchers have developed photothermal agents such as mesoporous Prussian blue NPs [80,81], Mo-based polyoxometalate clusters [28,82], PbS/CdS/ZnS quantum dots [26,83], and so on. To prepare multifunctional nanomedicines with good biocompatibility, high stability, high drug load, degradability in vivo, nontoxic side effects, and high photothermal-conversion efficiency, it is a popular trend to hybridize inorganic and organic materials [84]. However, developing the perfect photothermal agent mentioned above for nanomedicines, and in vivo toxicity and photothermal-conversion efficiency are the most important issues.

### 2.2. Photosensitizers as Carriers for Fabricating High Drug-Loading Nanomedicine

PDT has become an important method in preclinical research and clinical practice in tumor treatment because of its minimally invasive and local tumor-killing ability without damaging surrounding healthy tissues and cells. The laser, PS, and oxygen are three indispensable elements in PDT. An ideal PS has high absorption coefficient in the 650–850 nm region, high ^1^O_2_ yield, solubility under physiological conditions, high tumor selectivity, low damage to healthy tissues, and few side effects. However, the PSs developed at present have some disadvantages. Hematoporphyrin isolated from hemoglobin is the first-generation PS, which has obvious toxicity. The second-generation PSs such as Ce6 and methylene blue are mostly hydrophobic drugs, which easily aggregate in the aqueous medium, and have no tumor selectivity and poor bioavailability. Therefore, the third-generation PS combining PS with a nanosized delivery system or nanomedicines has been developed. Those nanoformulated PSs are also called smart PSs because of their specificity and good therapeutic effects [85,86]. Table 2 summarizes the different photosensitizers as carriers for fabricating high drug-loading nanomedicines.

#### 2.2.1. Inorganic Materials

In recent years, the development of inorganic material synthesis technology has been booming. Naturally, different kinds of inorganic materials with appropriate sizes and surface properties have been prepared and used as photosensitizers in the field of PDT. At present, the reported inorganic materials that can be equipped with chemotherapeutic drugs and enhanced PDT include CdSe/ZnS quantum dots [87], gold nanorods [88], reduced graphene oxide [89], and black phosphorus [90]. Those inorganic nanomedicines can not only directly generate singlet oxygen, but also change the microenvironment of tumor cells and promote the PDT effect.

**Figure 5:**
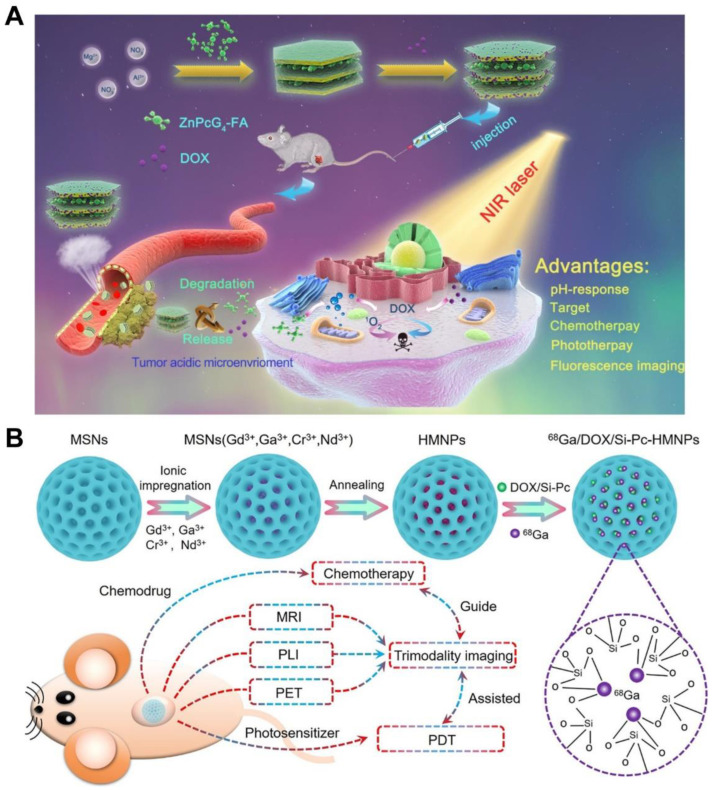
Preparation of high drug-loading nanomedicines by inorganic nanoformulas for chemo–photodynamic combination therapy of tumors. (**A**) Schematic illustration of the synthesis and antitumor performance of LDH_ZnPcG_4_-FA/DOX nanoplatform [91]. (**B**) Schematic illustration for the synthesis of ^68^Ga/DOX/Si-Pc-Loaded HMNPs [92]. Reprinted with permission from Ref. [91]. Copyright 2020, Elsevier. Reprinted with permission from Ref. [92]. Copyright 2021, American Chemical Society.

Wang et al. constructed novel stimulation-responsive multifunctional nanomedicines by inserting folate-linked ZnPcG_4_ molecules (ZnPcG_4_-FA) into layered dihydroxide (LDH) via a simple coprecipitate method [91]. Chemotherapy drugs (DOX) are loaded on the surface of LDH, and the loading efficiency of DOX was calculated to be 41.7% (Figure 5A). In the acidic tumor microenvironment, the release of DOX can be controlled with the degradation of LDH. The LDH is an interesting series of layered inorganic materials with the chemical formula as [M^2+^_−x_M^3+^_x_(OH)_2_]_x_^+^ A^n−^_x/n_·uH_2_O, where M^2+^, M^3+^ and A^n−^ represent divalent, trivalent metal ions and charge-balancing interlayer anion. Due to the special structure and pH response performance, LDH has been used as a carrier in drug delivery. Upon the 650 nm laser irradiation, the nanomedicines demonstrated excellent anticancer efficiency in vitro and in vivo because of the chemo–photodynamic combination therapy derived from the released DOX and ZnPcG_4_ photosensitizer.

**Table 2:** A summary of different PSs as carriers for fabricating high drug-loading nanomedicines.

Classes	Photosensitizer	Chemotherapeutic Drug	Nanoformulation	Drug-Loading Content (wt%)	Ref.
Inorganic	BODIPY	Doxorubicin	BODIPY-derivate MOFs	49.7%	[92]
	Si-Pc	Doxorubicin	Hybrid mesoporous NPs	DOX: 34.5%, Si-Pc: 51.2%	[93]
	PPa	Doxorubicin	UCNP@SiO_2_/PPa&DOX	DOX:72.8%	[94]
	Ir(III) complex	Cisplatin	Pt&Ir@polymer NPs	Pt: 38.9%, Ir(III): 12.9%	[95]
	Porphyrin	Doxorubicin	AuNP@dsDNA/Porphyrin	DOX: 75.0%	[96]
	PpIX	Doxorubicin	ZiF-67/8@DOX-PpIX NPs	DOX: 12.5% PpIX: 25.3%,	[97]
	UCNPs, Eosin Y	Camptothecin	UCNPs@CPT NPs	CPT: 53.7%	[98]
	Chlorin e6	Doxorubicin	rGO-DOX-Ce6 NPs	DOX: 82.3%	[99]
	TCPP	Doxorubicin	porphyrin MOFs	DOX: 52.2%	[100]
Organic	Chlorin e6	GA	GA-Ce6-FA NPs	Ce6 48.5%, GA 47.79%, FA 3.71%	[101]
	Chlorin e6	Cabazitaxel	LNA-CTX-Ce6 NPs	Cabazitaxel: 98.87%	[102]
	VPF	Doxorubicin	VPF-FRRG-DOX NPs	>70.0%	[103]
	Zn-TPPS	Doxorubicin	Zn-TPPS-HDP NPs	Zn-TPPS: 17.0%, DOX: 31.5%	[104]
	Zn-TPPS	Doxorubicin	H_2_TPPS@DOX NPs	DOX 42.4%	[105]
	TPCI	Paclitaxel	TPCI-PTX liposomes	PTX: 75.0%	[106]
	HPPH	Camptothecin	CPT-HPPH NPs	CPT: 55.0%, HPPH: 76.0%	[107]
	PpIX	Doxorubicin	DOX@PpIX-RGD NPs	DOX: 34.5%	[108]
	ALA	Doxorubicin	HA-chitosan@DOX-ALA NPs	DOX: 29.4% ALA: 11.5%	[109]
	PPA	Paclitaxel	PTX-PPA NPs	PTX: 44.2 %, PPA: 27.6 %	[110]
	PPA	Mitoxantrone	MTX-PPA NPs	MTX: 43.5%, PPA: 56.5%	[111]
	Chlorine e6	Doxorubicin	PEG-PBC-TKDOX@Ce6 NPs	DOX: 41.9%	[112]
	Chlorine e6	Paclitaxel	Ce6-PEG@PTX micelles	PTX: 90.1%	[113]
	TCPP	Doxorubicin	PEP FA@TCPP nanotubes	DOX: 30.5%	[114]
	Chlorine e6	Doxorubicin	DOX-NPs/Ce6-MBs NPs	DOX: 18.5%,Ce6: 67.1%	[115]
	TPC	Paclitaxel	RBC(M(TPC-PTX)) NPs	PTX: 38.0%, TPC: 13.0%	[116]

**Abbreviations:** BODIPY, dipyrromethene boron difluoride; PPa, Pyropheophorbide-a; PpIX, protoporphyrin IX; UCNPs, upconversion nanoparticles; CPT, camptothecin; rGO, reduced graphene oxide; TCPP, mesotetrakis(4-carboxyl)-21H,23H-porphine; GA, Gambogic acid; LNA, α-linolenic acid; VPF, Verteporfin; Zn-TPPS, tetra sodium meso-tetra (sulfonatophenyl)-porphyrin zinc (II); PTX, Paclitaxel; HPPH, photosensitizer 2-(1-hexyloxyethyl)-2-devinyl pyropheophorbide-a; TPCI, dual-functional theranostic PS; ALA, 5-aminolevulinic acid; PPA, pyropheophorbide a; MTX, mitoxantrone; PEP, peptide; TPC, 5,10,15,20-tetraphenylchlorin.

Recently, dipyrromethene boron difluoride (BODIPY) has been widely employed as an ideal photosensitizer for PDT, which is attributed to its strong absorption coefficient, significant photostability and high ROS yield. Meng et al. synthesized a novel two-fold interpenetration pillar-layered metal–organic framework (MOF) material (BBP-MOFs) using BODIPY-derivate bipyridine as a linker and photosensitizer, biphenyl-4,4′-dicarboxylic acid (BPDC), and Cd (NO_3_)_2_ [92]. BBP-MOFs are not only photodynamic agents that generate singlet oxygen (^1^O_2_) to perform PDT under 660 nm light irradiation, but also are drug carriers by loading DOX for chemotherapy. The results showed that BBP-MOFs exhibited high drug-loading efficiency of 49.7% as well as controlled drug release and ideal biocompatibility. 

Zou et al. designed and constructed multifunctional hybrid mesoporous nanomedicines (HMNPs) that integrate near-infrared persistent luminescence nanoparticle, magnetic nanoparticles (Gd_2_O_3_), and radionuclides (^68^Ga) via a large-pore (mesoporous silica nanoparticle) MSN-templated strategy (Figure 5B) [93]. The prepared HMNPs not only have good morphology, mesoporous structure, and surface properties, but also can load a variety of therapeutic agents at the same time. In the nanomedicines, the loading efficiency of DOX and photosensitizer (Si-Pc) were 34.5% and 51.2%, respectively. The studies on mice tumor models demonstrate that the DOX/Si-Pc-loaded HMNPs possess excellent cancer-cell-killing ability and an outstanding tumor suppression effect without systemic toxicity.

In addition to a variety of inorganic materials mentioned above, metal nanoparticles can be used in photodynamic therapy by conjugation or loading photosensitizer on their surfaces. For example, it is possible to modify the Au NPs either covalently or noncovalently with photosensitizer molecules [96,117]. Magnetic nanoparticles (MNPs) are one of the few inorganic materials approved by the FDA for therapeutic or imaging use in vivo. Photosensitizer molecules could be conjugated to MNPs for simultaneous magnetic resonance imaging and PDT [118,119]. Other carbon nanomaterials, including CNTs, graphene, and carbon dots, have also been used as carriers in PDT [120,121,122]. Moreover, upconversion nanoparticles (UCNPs) can emit high-energy light words under lower energy radiation, which has the advantages of better tissue penetration depth and higher photochemical stability. Therefore, nanomedicines of photosensitizer materials based on UCNPs have been gradually developed for photodynamic therapy [123,124,125].

#### 2.2.2. Organic Materials

Biodegradable organic-based nanomedicines have been widely used in drug delivery, including photosensitizers loaded with organic materials for photodynamic tumor therapy. For example, coupling or loading photosensitizer with poly(ethylene glycol) (PEG) [104,126], amphiphilic block polymers [112,127], polysaccharides [109,128], folic acid [111], peptides [108,113], liposomes [106,115], and polystyrene [122] have been studied. Phycocyanin (PC) is considered to be an effective natural photosensitizer, but it has not been well utilized due to its low biostability and intracellular accumulation. In order to overcome these limitations, nanosized PC particles (LAPC/DOX) were prepared by grafting lactonic acid (LA) and loading adriamycin (DOX). Compared with PC solution, the storage stability and photostability of PC particles are significantly improved, and the formation of nanomedicines further improves their biological stability [129]. Zhang et al. synthesized pH/reactive ROS dual-responsive PEG-DOX conjugate (labeled TPD) through acyl alkynylamine click reaction by PEG dipropiolate (PEGB), amine-terminated thioketal (TKL), and DOX [126]. The prepared amphiphilic TPD not only has high drug-loading efficiency for photosensitizer chlorine e6 (Ce6), but is also sensitive to acidic tumor microenvironment (TME) and ROS. Under laser irradiation, Ce6 produces abundant singlet oxygen (^1^O_2_), enabling programmable accelerated release of DOX and more Ce6 at tumor sites. Luo et al. prepared a prodrug nanoparticle (CDC NPs) by co-encapsulation of single thioether-linked dihydroartemisinin (DHA) dimer and Ce6, then stabilized by albumin-capturing maleimide- and hypoxia-sensitive 2-nitroimidazole-modified carboxymethyl chitosan [128]. DHA-S-DHA served as a ROS-responsive carrier for Ce6 and a chemotherapeutic drug. Upon laser irradiation, Ce6 could generate reactive oxygen species (ROS), which not only exerted the effect of the PDT but also broke the ROS-sensitive single thioether bridge in the dimeric prodrug DHA-S-DHA, thus accelerating the disassembly of the nanomedicines. Moreover, some researchers attempted to functionalize Ce6 with amino acid [130] and poly (amido amine) generation dendrimer [131] for better therapeutic effect.

The carrier-free nanomedicines, made up of photosensitizers and antitumor drug molecules, have become a kind of common nanomedicines for chemo–photodynamic combination therapy. Liu et al. fabricated porphyrin-based drug self-framed delivery systems without any carrier materials, in which water-soluble photosensitizer (tetra sodium mesotetra (sulfonatophenyl)-porphyrin, H2TPPS) and DOX could self-assemble to form H_2_TPPS@DOX nanomedicines by supramolecular interaction such as hydrophobic, electrostatic and π-π stacking interactions [105]. Owing to the higher drug-loading efficiency of 42.4%, the H2TPPS@DOX could effectively generate singlet oxygen to obviously block DOX efflux and ultimately induce apoptosis to effectively reverse multidrug resistance of tumor cells under light irradiation.

## 3. High Drug-Loading Nanomedicines for Chemo–Photothermal Combination Therapy

Recently, increasingly more attention has been paid to the integration of chemotherapy and photothermal (PTT) on the same platform for a combination therapy, which may open up new avenues for noninvasive tumor treatment in the future. The combination of PTT with chemotherapy has the following advantages. (1) PTT, as a fast and efficient tumor treatment, can directly kill tumor cells through local heating to make up for the slow onset of chemotherapy. (2) Owing to the changes in vascular permeability, blood flow, and tumor oxygenation caused by the increase in tissue temperature, PTT may make tumor cells more sensitive to chemotherapy and other treatments. (3) Chemo–photothermal combination therapy may overcome the multidrug resistance of current chemotherapy. The combination of these two strategies has shown significant therapeutic effects, but the significant synergetic effect rather than just mixing two treatments is important to be demonstrated. Although there are many benefits of this combination for chemotherapy, the drug-loading efficiency of nanomedicines determines the number and dose of administration to some extent. To achieve the ideal therapeutic effect, low drug-loading nanomedicines often need to be increased in dosage and frequency of administration, which also increases the risk of toxic and side effects. However, at present, researchers are paying less attention to the drug-loading efficiency of nanomedicines in the treatment of chemotherapy/PTT combination therapy.

**Figure 6:**
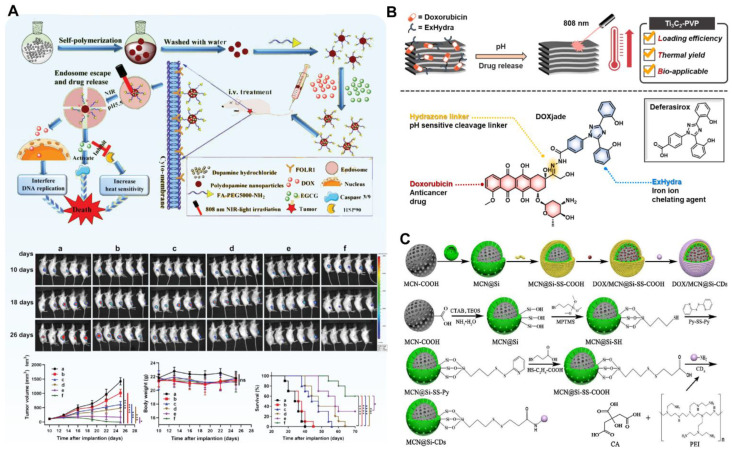
(**A**) Schematic illustrating the formation of the DOX-EGCG/PDA-FA NPs [133]. The representative tumor growth was shown in vivo by bioluminescence imaging at 10, 18, and 26 days after implantation. Changes in tumor volume, body weight and survival curves of mice given different treatments. (**a**) Normal saline (control), (**b**) free DOX, (**c**) DOX/DPA NPs+laser, (**d**) DOX-EGCG/DPA NPs+laser, (**e**) DOX/DPA-FA NPs+laser, (**f**) DOX-EGCG/DPA-FA NPs+laser. * *p* < 0.05, *** *p* < 0.001, and **** *p* < 0.0001 compared to f (n = 10) (**B**) Schematic representation of DOX jade being loaded onto 2D ultrathin Ti_3_C_2_ MXene nanosheets to create constructs that allow for combined iron chelation chemo–photothermal therapy [134]. (**C**) Schematic illustration of the formation of DOX/MCN@Si-CDs and MCN@Si-CDs [135]. Reprinted with permission from Ref. [133]. Copyright 2021, John Wiley & Sons, Inc. Reprinted with permission from Ref. [135]. Copyright 2020, Elsevier.

The target of antitumor drug molecules is in the cell, so effective endocytosis and intracellular drug release are the basis for the high-efficiency, antitumor effect of nanomedicines. Nanoparticles generally rely on endocytosis to enter cells through cell membrane-bound vesicles, and the mechanism of receptor ligand-mediated endocytosis is the clearest [132]. When the specific receptor on the surface of the cell membrane meets with its corresponding ligand, the two bind to form a receptor ligand complex, and then through the invagination of the cell membrane, the integument pits are formed, and the integuments are further formed to enter the cytoplasm. Fan et al. developed tumor acidity and NIR-responsive folic acid (FA) functionalized PDA NPs and loaded DOX and EGCG for chemo-PTT combination therapy (Figure 6A) [133]. The drug is loaded into PDA NPs through π-π stacking and hydrogen-bond interaction, and the total drug load exceeds 30%. Based on this design, DOX-EGCG/PDA-FA NPs release drugs in response to the acidic tumor environment and NIR laser stimulation. In addition, DOX-EGCG/PDA-FA NPs combined with FA receptors highly expressed on the surface of tumor cells can improve the drug internalization rate of cells. A biodistribution study in vivo indicates that PDA-FA NPs can enhance tumoral accumulation and display 19-fold higher intratumoral distribution than free drug groups at 24 h postinjection. In vivo treatment experiments show that 60% of breast tumor-bearing mice survive over 70 days in the DOX-EGCG/PDA-FA NP group (Figure 6A(a–f)). Xu et al. constructed AuNBs@PDA/DOX nanocomposites by coating the surface of Au NBs with PDA and loading DOX through π-π stacking and hydrogen bonding between DOX and PDA [23]. The nanocomposites have strong absorption in the NIR region, and their photothermal-conversion efficiency (75.48%) is higher than that of Au nanospheres. The drug-loading efficiency of DOX in AuNBs@PDA nanocomposites can reach about 70%, and has a high release rate under a low pH environment and NIR laser irradiation. In vitro and in vivo studies have shown that AuNBs@PDA/DOX nanocomposites have a significant antitumor effect, and the side effects are negligible.

Ti_3_C_2_ MXene nanosheets are a new type of 2D material showing many advantages in biomedical applications of nanomedicines, including significant light-absorption capacity under NIR light, large specific surface area, and good stability in air and solution. Xu et al. conjugated DOX and iron chelator (ExJade^®^) to form prodrug molecules and then loaded the conjugate (DOXjade) onto ultrathin 2D Ti_3_C_2_ MXene nanosheets, producing a dual-therapeutic prodrug nanomedicine (Ti_3_C_2_-PVP@DOXjade) involving iron chelation chemo–photothermal combination therapy (Figure 6B) [134]. Two-dimensional ultrathin Ti_3_C_2_ Mxene nanosheets are expected to have strong interactions with DOXjade owing to the planar structure with a large surface area. The drug loading of Ti_3_C_2_-PVP@DOXjade is 67.74%. DOXjade’s iron chelation and chemotherapy functions are photoactivated at the tumor site, while enhancing the powerful photothermal effect with a photothermal-conversion efficiency of up to 40%. A tumor pH-responsive iron chelation/photothermal/chemotherapy antitumor effect is achieved both in vitro and in vivo. Li et al. used soybean phospholipid-stabilized 2D Ti_2_N nanosheets to load DOX, coated biodegradable silica shell, and loaded CDDP to construct multifunctional Ti_2_N@oSi for drug-delivery control and collaborative antitumor therapy [77]. The obtained Ti_2_N@oSi nanomedicines show salient photothermal-conversion efficiency (41.6%), ultrahigh drug loading (88.8%), dual drug loading, pH/glutathione (GSH)/photothermal-responsive drug-release dynamics, and targeting ability for tumor cells. The imine bonds between CDDP and the organosilica shell can be broken in the acid environment at tumor sites, resulting in the pH-responsive release of CDDP from the Ti_2_N@oSi nanocarriers. In addition, the high intracellular GSH level of tumor cells can break the disulfide bond in the organosilica shell, leading to the collapse of the organosilica shell and the depletion of GSH, thereby increasing the chemosensitivity of tumor cells and triggering the release of DOX. Mesoporous or hollow structure materials are often used to prepare nanomedicines with high drug loading due to their larger storage space. Wang et al. compared the NIR photothermal effect of mesoporous carbon nanospheres (MCNs) and GO nanosheets, as well as the drug load and drug-release behavior of the model drug DOX [34]. The experimental results show that MCN-PEG exhibits better photothermal stability and elevated temperature than GO-PEG under multiple NIR irradiation in vitro and in cells. The loading efficiency of DOX/MCN-PEG and DOX/GO-PEG is 69.2% and 68.3%, respectively. MCNs have slightly higher drug-loading efficiency owing to their ordered mesoporous channels and high surface area. Lu et al. prepared a traceable dual-porous mesoporous silica-coated mesoporous carbon nanocomposite (MCN@Si) with high drug-loading efficiency (48.2%) and high photothermal-conversion efficiency (30.5%) (Figure 6C) [135]. Connecting carbon dots (CDs) with disulfide bonds on the surface of MCN@Si blocks the mesopore and prevents premature release of DOX in DOX/MCN@Si-CDs. Additionally, CDs act as fluorescent probe, thereby providing the visualization potential of this nanomedicine. Huang et al. used Ag as a precursor to prepare hollow mesoporous Bi nanocapsules through metal replacement and then modified them with thiol-PEG-folate acid to obtain a new dual-stimuli-responsive single-“elemental” photothermal nanomedicines (HM-Bi@PEG-FA NSs) for combination chemo-PTT therapy of tumor [47]. The designed hollow mesoporous nanomedicine has excellent photothermal-conversion ability (34.72%) and high loading efficiency of DOX (78%). In vitro and in vivo tumor therapies were performed, demonstrating effective tumor inhibition and ablation through combination of chemo-PTT. Wu et al. synthesized hollow copper sulfide NPs (HCuSNPs) and developed a melanoma cell (B16F10 cell) membrane-encapsulated DOX and ICG-loaded hollow copper sulfide NPs (IDHCuSNP@B16F10) for targeted PTT, photoacoustic imaging, and chemotherapy [76]. The hollow mesoporous structure of HCuSNP makes it an ideal drug carrier. The loading efficiency of ICG and DOX in IDHCuSNP@B16F10 are as high as 98% and 85%, respectively. IDHCuSNP@B16F10 has an obvious homologous targeting effect and shows good, combined chemo-PTT in animal models of melanoma.

Carrier-free nanodrugs are a kind of nanomedicine composed entirely or mostly of self-assembled active pharmaceutical ingredients without exotic nontherapeutic carriers. They are attracting considerable attention because of their high drug loading and excellent performance. Lu et al. developed three-component nanoparticles, Ang-PEG-g-PLL@CPT-RT@IR783, which first self-assemble into stable two-component NPs, CPT-RT@IR783, by coupling amino acids to camptothecin (CPT) and an NIR dye (IR783). Then, the positively charged Ang-PEG-g-PLL was coated onto the negatively charged two-component NPs by electrostatic interaction [39]. The carrier-free nanocomplex exhibited a high drug-loading efficiency (up to 62%), good biocompatibility, and effective glioma-accumulation ability. In vitro and in vivo results show that the Ang-PEG-g-PLL@CPT-RT@IR783 NPs can effectively pass the blood–brain barrier and target glioma cells. Moreover, for the treatment of chemo–photothermal combination therapy, the nanocomposite has a better therapeutic effect, longer survival time, and minimal toxic side effects in orthotopic glioblastoma tumor-bearing nude mice.

## 4. High Drug-Loading Nanomedicines for Chemo–Photodynamic Combination Therapy

The combination of PDT and chemotherapy shows great potential in preclinical studies and has been gradually applied in clinical tumor treatment. The chemotherapeutic drugs and PSs need to enhance the specific therapeutic effect on the tumor site, so drugs targeting the tumor site at the same time are particularly important. In this context, the reasonable design and synthesis of nanomaterial-based carriers have become the key to nanotechnology. To pursue higher therapeutic efficiency, obtaining high drug-loading nanoparticles for chemo-PDT combination therapy is feasible by loading PSs and chemotherapy drugs simultaneously on a single nanoplatform.

Lan et al. developed novel carrier-free nanomedicines (GA-Ce6-FA NPs) by using gambogic acid (GA), chlorin e6 (Ce6), and folic acid (FA) in a simple green self-assembly way to realize the combined treatment of chemotherapy and PDT [101]. GA in GA-Ce6-FA NPs is a chemotherapeutic drug that can destroy cell redox homeostasis, consume GSH in tumor, increase ROS, and enhance the efficacy of PDT as well. The drug load of the three components in the optimized NPs is 48.5% Ce6, 47.79% GA, and 3.71% FA, suggesting a higher drug-loading ratio than traditional nanoscale drug-delivery systems. GA-Ce6-FA NPs have pH-triggered drug-release characteristics under weakly acidic conditions and are able to quickly release drugs in tumor areas. In vivo treatment experiments have shown that the tumor weight of GA-Ce6-FA NPs irradiated by laser is the lowest with TIR of 88.2%, with no obvious toxicity found. Thus, the clearance of GSH by chemotherapeutic drugs is an effective way to improve the efficacy of PDT.

**Figure 7:**
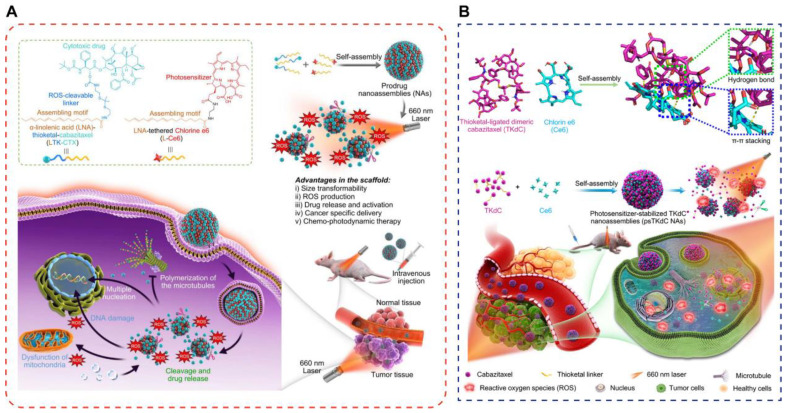
(**A**) Schematic illustrating photoactivatable self-assembling prodrug cocktail (PSPC) nanomedicines constructed from small molecules for tumor-specific drug activation and combined photodynamic therapy and chemotherapy [102]. (**B**) Rational design of photosensitizer-stabilized, self-assembling, supramolecular nanotherapy (psTKdC NAs) for combination chemo-phototherapy [136]. Reprinted with permission from Ref. [102]. Copyright 2021, Elsevier. Reprinted with permission from Ref. [136]. Copyright 2020, Elsevier.

Huang et al. proposed a simple and universal strategy to construct photoactivated self-assembled prodrug cocktail nanomedicines for the specific drug activation and chemo-PDT of tumors (Figure 7A) [102]. In other words, the chemotherapeutic drug cabazitaxel (CTX) and Ce6 were pufayized by polyunsaturated fatty acids (PUFA), and the two pufayated prodrugs were self-assembled into cocktail nanomedicines without any exogenous excipient. The drug-load rates for CTX and Ce6 were exceptionally high as 98.87% and 98.95%, respectively, and the total drug-load content was determined to be 64.65%. Under NIR light irradiation, the PS produces ROS, which spontaneously degrades the thioketone bond and activates the cytotoxic drug, carbataxel. Thus, light and tumor-specific cascades simultaneously produce phototoxicity, thereby accelerating the release of carbataxel and enhancing the therapeutic combination effect on cancer.

The clinical translation of nanomedicines for combination therapy that can facilitate multimodal therapies remains a considerable challenge. Self-assembled small-molecule NPs with the characteristics of NIR photoresponsive drug activation, size conversion, combination synergy, and significantly reduced toxicity were constructed by Huang et al. [136]. Their approach was based on the ROS-activatable thioketal linkage of the anticancer CTX drug to generate the dimeric TKdC prodrug, which subsequently coassembles with Ce6 (termed psTKdC NAs) (Figure 7B). The nanoassemblies achieve nearly quantitative drug-load efficiency and exceptionally high drug loading (>90%). Upon NIR laser irradiation, psTKdC NAs are transformed into smaller size particles and facilitate the production of pharmacologically active CTX.

Wang et al. developed a dual-function therapeutic PS, namely, TPCI, which has a surprising ^1^O_2_ quantum yield of 98.6% in water and can simultaneously self-report PDT treatment response from the beginning of treatment [137]. In their previous study, using TPCI as a PS successfully inhibited tumor growth, with an initial size less than or equal to 100 mm^3^. However, when treating larger tumors, PDT results with TPCI were significantly impaired. Subsequently, they constructed an ultra-efficient chemotherapy–PDT platform (TPCI/PTX@Lipo) by co-coating liposomes with the TPCI and chemotherapy drug paclitaxel [96]. The liposomes also show excellent drug-loading efficiency with over 75% encapsulation efficiencies. In vivo experiments show that the tumors with an initial size of about 200 mm^3^ are almost completely eliminated by TPCI/PTX@Lipo after irradiation, which is better than many previously reported PDTs that can only inhibit tumor growth but cannot directly ablate tumors. Jiang et al. designed and synthesized an ROS-activatable heterodimeric prodrug (named HRC) for tumor-selective imaging, chemo–photodynamic combination therapy [107]. The prodrug comprises camptothecin (CPT), the PS 2-(1-hexoxyethyl)-2-devinyl pyropheophorbide-a (HPPH), and a thioketone linker. Compared with CPT- or HPPH-loaded polymer NPs, HRC-loaded NPs have higher drug-load efficiency (over 95%), better colloidal stability, and less premature drug leakage. Interestingly, HRC NPs have almost no fluorescence owing to strong π-π stacking and could be effectively activated by endogenous ROS once entering cells. Considering that the ROS level in cancer cells is higher than that in normal cells, HRC NPs can selectively light up cancer cells and have stronger cytotoxicity to cancer cells. Furthermore, owing to its unique characteristics, inorganic materials are often used as nanocarriers to load chemotherapeutic drugs and PSs for combined chemotherapeutic and PDT of tumors. Jiang et al. prepared the upconversion nanomaterial NaGdF_4_:Yb,Er@NaGdF_4_:Yb,Nd (abbreviated as UCNP) with core–shell structures [94]. Then, a thin layer of mesoporous silica is coated onto the UCNP surface to obtain UCNP@SiO_2_, and the PS PPA and DOX are filled into the pores of the SiO_2_ layer to obtain UCNP@SiO_2_/PPA&DOX. The loading efficiency of DOX by the NPs is as high as 72.8%. Cui et al. prepared Au NP-DNA based nanomedicines by nontemplated extension of short DNA primers on Au NP core through terminal deoxynucleotidyl transferase catalysis and subsequent DNA hybridization to realize the synergistic loading and delivery of DOX and porphyrin/G-quadruplex composited PS [96]. The DNA-based nanomedicines have high loading efficiency (75%) and tumor-targeted accumulation of DOX and porphyrin/G-quadruplex composite PS and show rapid payload release in a stimulus-responsive mode.

## 5. High Drug-Loading Nanomedicines for Chemo–Photothermal–Photodynamic Combination Therapy

To completely kill tumor cells in a diversified and cooperative way at the early stage, the design and construction of a nanomedicine system that can play a role in multimode collaborative therapy is the current research goal. PTT and PDT have the advantages of minimal invasiveness, local treatment, rapid onset, and good efficacy in a short time. However, the shortcoming is that the laser cannot completely cover the tumor tissue in many cases, and the penetration depth is limited, resulting in incomplete treatment and tumor edge recurrence. Chemotherapy is a systemic tumor-treatment method with the disadvantage of being highly toxic and having side effects, but the advantage is that small molecule drugs can penetrate deep tumor tissues and marginal tissues for a long time. Therefore, chemotherapy, PTT, and PDT can make up for each other’s shortcomings, and the combination of the three can exert a highly effective therapeutic effect. At present, many groups have made great efforts to develop various chemo–photothermal–photodynamic combination therapy nanomedicines. Among them, some have high drug-loading efficiency through elaborate design and fine control. Table 3 summarizes the chemo-PDT-PTT combination therapy with common photothermal agents and PSs used as carrier materials.

**Figure 8:**
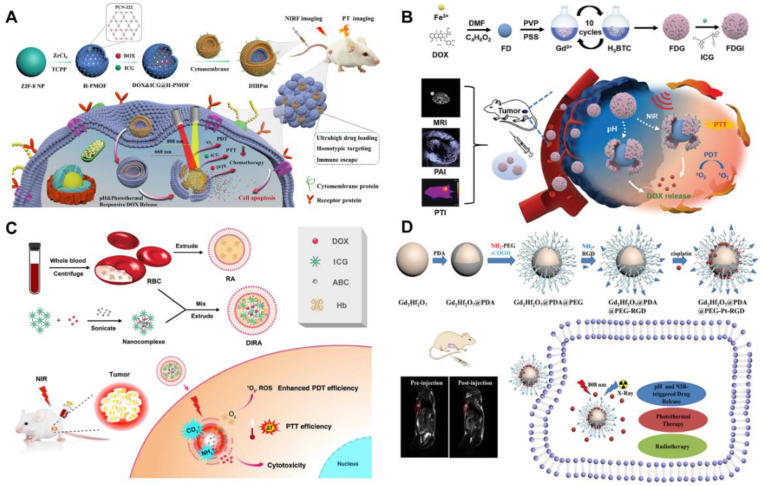
(**A**) Schematic illustration of fabricating the H-PMOF NPs through a self-sacrificial ZIF-8 NP template strategy and schematic demonstration of the use of DIHPm for imaging-guided synergistic PDT/PTT/chemotherapy of tumor [139]. (**B**) Schematic illustration of the fabrication process of Fe-DOX@Gd-MOF-ICG, and the nanoplatform for MR/PA/PT imaging-guided chemo/PDT/PTT compound antitumor therapy [140]. (**C**) Illustrations for preparation of DIRAs and their combination effects against breast cancer by combining PTT/PDT and chemotherapy [142]. (**D**) Schematic illustration of the design and synthesis of Gd_2_Hf_2_O_7_@PDA@PEG-Pt-RGD nanomedicines for MRI-guided combined chemo/PDT/radiotherapy [146]. Reprinted with permission from Ref. [139]. Copyright 2021, American Chemical Society. Reprinted with permission from Ref. [146]. Copyright 2020, American Chemical Society. Reprinted with permission from Ref. [140]. Copyright 2020, John Wiley & Sons, Inc. Reprinted with permission from Ref. [142]. Copyright 2018, Elsevier.

MOFs are often selected as nanocarriers to load chemotherapeutic drugs and phototherapeutic agents because of their large drug loading, easy modification, and PDT properties. Doping Mn^2+^ in MOFs prepared with PS porphyrin as ligand can effectively promote the catalytic decomposition of H_2_O_2_, alleviate tumor hypoxia, and effectively provide oxygen for PDT under laser irradiation. Feng et al. chelated Mn^2+^ with tetrakis (4-carboxyphenyl) porphyrin (Mn-TCPP) to obtain MOFs with catalase properties [138]. Based on the porous structure of MOF, hydrophobic chemotherapeutic drugs (Iniparib) were successfully loaded, and PDA-modified HA was coated onto the surface of NPs to prepare a tumor-targeted chemo–photothermal–photodynamic combination therapy platform. They found that when the ratio of MOF to Iniparib (*w*/*w*) ranges from 1/1 to 1/10, the drug-loading efficiency increases from 6.54% to 42.54%. As systematically demonstrated in vitro and in vivo experiments, this nanotherapeutic approach enables the combined therapy with great inhibition on the tumor. Sun et al. fabricated hollow porphyrin MOF (H-PMOF) NPs with mesoporous spherical shells through a simple self-sacrificing ZIF-8 NP template strategy (Figure 8A) [139]. Compared with nonhollow porphyrin MOF NPs, the H-PMOF nanoplatform has a significantly enhanced photodynamic therapeutic effect and can be used as an excellent drug carrier, co-loaded with DOX and ICG, with an ultra-high drug loading of 86.4%. In vitro and in vivo studies have shown that this nanomedicine has combination chemotherapeutic/PDT/PTT anticancer activity under imaging guidance, and its systemic toxicity is negligible. Zhu et al. synthesized a biological MOF by the self-assembly of Fe^3+^ ions and DOX molecules. Then, other biological MOFs structure comprising Gd^3+^ ion and 1,3,5-phenyltricarboxylic acid (H_3_BTC) are wrapped on the surface of Fe-DOX NPs through a step-by-step assembly method. The PS ICG is adsorbed to prepare the chemotherapy/PDT/PTT multifunctional treatment platform (Fe-DOX@Gd-MOF-ICG) (Figure 8B) [140]. Considering that DOX molecules directly act as the organic ligands of MOF structures, Fe-DOX has a significant high drug loading of about 71.4%. The in vitro and in vivo outcomes indicate that the Fe-DOX@Gd-MOF-ICG nanoplatform has outstanding combination antitumor performance through magnetic resonance/photoacoustic/photothermal imaging-guided combined chemo-PTT/PDT. Yang et al. synthesized an upconversion NP-encapsulated MOF nanoplatform (UCNPs@MIL-100 (FE) NPs) loaded with DOX based on the porous structure and high specific surface area of MOFs (72% loading efficiency) [141]. In vivo and in vitro experiments show that the loaded DOX UCNPs@MIL-100(Fe) nanomedicines significantly inhibit tumor cell growth based on chemotherapy/PDT/PTT combination effects under very mild 808 nm laser irradiation (0.5 W/cm^2^).

ICG is a phototherapeutic agent that can be used in PTT and PDT. It shows a strong antitumor effect when used in combination with the chemotherapeutic drug DOX. Wan et al. prepared nanoscale red blood cells (RAS) containing sufficient oxygen Hb and the gas-producing agent ammonium bicarbonate for the co-loading and controlled release of ICG and DOX. The ICG and DOX encapsulation rates in nanomedicines reached 93.6% and 95.3%, respectively (Figure 8C) [142]. Tang et al. synthesized a low-cytotoxicity carboxylated polyamine (PAMAM) as a nanocarrier for loading the chemotherapeutic drug temozolomide (TMZ) and ICG [143]. The drug loading of TMZ and ICG was 44.91% and 99.87%, respectively. In vitro and in vivo experiments show that the combined application of NPs loaded with TMZ and ICG can kill melanoma cells and inhibit their growth after NIR-light irradiation. Shi et al. also prepared nanomedicines targeting mitochondria by loading DOX and ICG with hollow mesoporous silica NPs modified by the phase-change material L-menthol (LM) for the chemo-PTT/PDT combination therapy of tumors [144].

The strategy of combining imaging and treatment is a widely studied and promising approach. Xu et al. integrated copper sulfide (CuS) NPs and black phosphorus (BP) nanosheets on the mesoporous silica coated transformation NPs (UCNPs) and then loaded DOX for the combination treatment of tumors by PTT, PDT, and chemotherapy [145]. In this system, the pore size of mesoporous silica and the large specific surface area of BP were conducive to obtain a high DOX loading efficiency (77.4%). Magnetic resonance imaging (MRI) and computed tomography induced by rare-earth-ion doping enable the nanomedicines to have multimode imaging ability under 808 nm light irradiation and thus achieve image-guided tumor treatment. Kuang et al. designed a kind of nanoparticles by loading Gd_2_Hf_2_O_7_ NPs and being modified with PDA, PEG, and c(RGDyK) peptide (RGD) for combined chemotherapy/PTT/radiotherapy of drug-resistant tumors (Figure 8D) [146]. Based on the high drug-loading efficiency of PDA, the load amount of CDDP in the nanomedicines is 33.8%. The nanomedicines have good NIR-absorption capacity and high X-ray attenuation efficiency. They show a potential photothermal effect under 808 nm NIR laser irradiation, significantly improving ROS generation after X-ray radiation. Wang et al. synthesized BP quantum dots (BPQDs) and functionalized them to load DOX for chemo-PTT/PDT combination tumor therapy [147]. BPQD is a metal-free layered semiconductor and has a higher surface-to-volume ratio than other 2D materials, such as selenium, transition-metal disulfide, and graphene. This property of BP increases its drug-loading efficiency. In this study, FA-PEG@BPQD@DOX was prepared with a drug-loading efficiency of 65% and is able to achieve precise three-mode collaborative therapy at the tumor site by guiding nanomedicines through a visual image.

**Table 3:** A summary of high drug-loading nanomedicines for chemo–photothermal–photodynamic combination therapy.

Nanoformulation	Photothermal Agent	PS	Chemotherapeutic Drug	Drug-Loading Content (wt%)	In Vitro/In Vivo; Biological Model	Findings	Ref.
Bi_2_WO_6_-DOX-PEG NPs	Bi_2_WO_6_ NSs	Bi_2_WO_6_ NSs	DOX	81.3%	In vivo and in vitro; U14 cells model	Bi_2_WO_6_ nanomaterials can produce ROS and high hyperthermia effect under low power density NIR light irradiation.	[20]
rGO-PEG-DOX-Ce6 NPs	rGO	Ce6	DOX	DOX:82.3% Ce6:92.5%	In vivo and in vitro; U87 cells model	PTT was more effective than chemotherapy and PDT in the 3D spherical model of tumors.	[99]
Ini@PM-HA-PDA NPs	PDA NPs	Mn-TCPP MOF	Iniparib	42.54%	In vitro and in vivo; MDA-MB-231 cells model	Biodegradable NPs for oxygen production was constructed to enhance PDT/PTT	[138]
(DOX and ICG)@H-PMOF@mem NPs	ICG	H-PMOFs	DOX	86.4%	In vitro and in vivo; 4T1 cells model	The NPs with mesoporous spherical shells were capable of pH control and DOX release triggered by near-infrared laser.	[139]
Fe-DOX@Gd-MOF-ICG NPs	ICG	ICG	DOX	71.4%	The in vitro and in vivo; 4T1 cells model	The MOFs with magnetic resonance imaging and controlled drug release were synthesized.	[140]
UCNPs@MIL-100(Fe)-DOX NPs	MIL-100(Fe)	UCNPs	DOX	72.0%	In vivo and in vitro; U14 cells model	MOFs coated UCNPs were synthesized by a facile one-pot liquid-solid-solution method.	[141]
ICG/DOX co-loaded RBCs	ICG	ICG	DOX	95.5%	In vitro and in vivo; 4T1 cells model	Red blood cells containing oxyHb and NH_4_HCO_3_ were prepared for co-loading and controlled release of ICG and DOX.	[142]
PAMAM-TMZ ICG@HA NPs	ICG	ICG	TMZ	45.2%	In vitro and in vivo; A375 cells model	A low cytotoxic carboxyl polyamine was synthesized as a nanocarrier.	[143]
THMSNs@LM-DOX-ICG NPs	ICG	ICG	DOX	37.3%	In vitro, HeLa cells	A smart subcellular organelle was designed as an effective drug delivery platform.	[144]
BP/UCNP-SiO_2_-CuS-PEG-DOX NPs	BP, CuS NPs	BP, CuS NPs	DOX	77.4%	In vitro and in vivo; U14 cells model	The reduction in the red/green (R/G) ratio elicited by DOX release can be employed to determine the extent of DOX release.	[145]
BPQDs-PEG-DOX NPs	BPQDs	BPQDs	DOX	65%	In vitro and in vivo; HEK 293T cells model	The nanoplatform can inhibit tumor growth through visualized synergistic treatment and photoacoustic and photothermal imaging	[147]
Fe_3_O_4_@PDA/PEG/ICG-DOX NPs	PDA NPs	ICG	DOX	50.0%	In vitro; HeLa cells	Facilitating cell internalization of drugs under a localized magnetic field.	[148]
5-FU/rGO/Bce hydrogel	rGO	Bce	5-FU	48.4%	In vitro; HeLa cells	Bce, a PS, participates in hydrogel crosslinking and improves biocompatibility.	[149]
BP-DOX nanosheets	BPs	BPs	DOX	90.5%	In vivo and in vitro; 4T1 cells model	BP nanosheets as a multimodal treatment platform for cancer treatment.	[150]
Fe_3_O_4_@MnO_2_@PPy-DOX NPs	PPy, Fe_3_O_4_	PPy, MnO_2_	DOX	70.0%	In vitro; HepG2 cells model	A nanocomposite with Fe_3_O_4_ as core and two layers of MnO_2_ and PPy as the shell was prepared to enhance PDT/PTT.	[151]
Pa-Hyd-DOX NPs	Pa	Pa	DOX	53.1%	In vivo and in vitro; SCC25 cells models	Greatly promoted tumor penetration and cell internalization.	[152]
IONCs@Ce6-DOX/PCM NPs	IONCs	Ce6	DOX	41.2%	In vitro and in vivo; HeLa cells model	The designed nanomedicine can realize the combination therapy triggered by single light.	[153]
P(DPP-BT/DOX) NPs	DPP	DPP	DOX	45.7%	In vitro and in vivo; HeLa cells model	The newly synthesized small-molecule dye shows strong absorption in the NIR-I and fluorescence emission in the NIR-II.	[154]
Mn@Au@TiO_2_@DOX NPs	Au@TiO_2_ NPs	Au@TiO_2_ NPs	DOX	45.5%	In vitro and in vivo; HeLa cells model	The Au@TiO_2_ core-shell NPs showed stronger photodynamic properties than commercial TiO_2_ and Au/TiO_2_ composites.	[155]
MXene-DOX	Ti_3_C_2_ nanosheets	Ti_3_C_2_ nanosheets	DOX	87.3%	In vitro; Hela cell	Ti_3_C_2_ nanosheets were processed into three-dimensional honeycomb structures with anti-aggregation properties as nanocarriers.	[156]
5-Fu@ICG-PNIPAM nanogels	ICG	ICG	5-Fu	76.7%	In vitro; Hela cell	The nanogels improved drug bioavailability and achieved controlled release.	[157]

**Abbreviations:** PDA, poly-dopamine; rGO, reduced graphene oxide; 5-FU, Fluorouracil; TMZ, temozolomide; Bce, Brassica chinensis extract; BPs, black phosphorus, PPy, polypyrrole, Pa, pheophorbide a; IONCs, iron oxide nanocrystals, DPP-BT, diketopyrrolopyrrole -based small-molecule dye; BPQDs, black phosphorus quantum dots, Iniparib, Poly (ADP-ribose) polymerase (PARP) inhibitor; H-PMOF, hollow porphyritic metal–organic framework; 4T1 cell, mouse breast cancer cell; A375 cell, human melanoma cell; U14 cell, murine cervical cancer cell; SCC25 cell, human oral cancer cell; U87 cell, human glioma cell; HepG2 cell; human hepatoma cell; HEK 293T cell, human embryonic kidney cell; MDA-MB-231 cell, human breast cancer cell.

## 6. Discussion and Perspectives

Nowadays, the combination of phototherapy and chemotherapy is a useful way to treat tumors effectively and safely. The high drug-loading nanomedicines associated with this method have become a potential treatment in clinics. However, the research remains limited, while ordinary high drug-loading nanomedicines are difficult to meet the requirements of tumor chemo–photo combination therapy. Compared with traditional single phototherapy or chemotherapy, high drug-loading nanomedicines for combination therapy show many advantages, such as improved tumor targeting, improved drug bioavailability, reduced toxicity, flexible controlled drug release, and enhanced combination of therapeutic effects.

### 6.1. Discussion and Challenges

Although these nanomedicines have shown exciting results in the laboratory or preclinical animal studies, they still face many challenges as follows:

(1) High drug-loading nanomedicines use as little carrier material as feasible, yet most nanomedicines still cannot be independent from the carrier. Therefore, the potential safety of carrier materials is one of the most crucial issues, especially those non-biodegradable inorganic materials, which will stay in the body for a long time after being consumed. The Au NPs may be promising in this field for their initial success in clinical trials. However, there has not been any systematic research on how to choose suitable and secure carrier materials to build high drug-loading nanomedicines.
(2) Most current studies have concentrated primarily on the design of nanomedicines, but little research compares high and low drug-loading nanomedicines on the internalization mechanism, intracellular release, in vivo circulation time, and so on. For nanomedicines with drug-loading content higher than 50%, their sustained-release characteristics may be far lower than expected, while the pharmacokinetic studies in such studies are often lacking. Hence, it is urgent to conduct the research on the above-mentioned factors, which are of great significance to promote the clinical application of high drug-loading nanomedicines.
(3) When constructing high drug-loading nanomedicines using photothermal materials and photosensitizers as nanocarriers, it is crucial to strike a balance between the phototherapeutic efficacy and the amount of carrier. Although this paper reviews the typical photothermal materials and novel photosensitizers employed as the carrier to manufacture high drug-loading nanomedicines in recent years, there is still a lack of systematic theories and techniques in this area. The construction of such nanomedicines is dependent on the knowledge of predecessors, and needs to be further explored and summarized.
(4) Although most of the chemo–photo combination therapy nanomedicines reported so far show combination antitumor effects, whether two or three treatment modes maximize combination efficiency is uncertain. The combination therapy theory between chemotherapy and phototherapy is not clear at present, and the specific combination mechanism has not been clearly explained. Professional methods and guidance for the calculation of combination efficiency between chemotherapy and phototherapy, as well as a programmed design in this aspect, remain lacking. Moreover, given that the design of those nanomedicines is usually uneconomical or impossible through empirical methods, mathematical and computational modeling can serve as a powerful tool to allow for the controlled study of these processes [158].
(5) At present, PTT and PDT have been employed in clinical treatment trials of some tumors, including basal cell carcinoma, in situ squamous cell carcinoma [159], esophageal cancer, colorectal cancer, [160] and high-grade glioma [161]. The results indicated that the treatment group achieved better antitumor effect compared with the control group and placebo group. However, the limited light penetration depth hinders the clinical application of phototherapy. Nonuniform irradiation of tumor tissue and insufficient irradiation depth make it impossible to completely eliminate the tumor cell for phototherapy, leading to following tumor recurrence and metastasis. Fortunately, chemotherapy and phototherapy can make up for each other’s shortcomings. Chemotherapeutics have a longer effect time and are more evenly distributed in the tumor tissue. After phototherapy rapidly and efficiently kills tumor cells, chemotherapy can eliminate the residual tumor cells for a long time. Therefore, chemo–phototherapy can significantly improve the treatment efficiency. Nowadays, physical therapies such as ultrasound therapy and magnetic hyperthermia are also applied in cancer treatment because of their better penetration depth. However, there are some problems in ultrasonic therapy, such as local overheating and inability to treat intestinal tumors. For magnetic hyperthermia, the large dosage of magnetic nanoparticles is often needed to achieve an ideal therapeutic effect, and the metabolism of those magnetic nanoparticles in vivo needs to be considered. In addition, such treatments require more expensive instrument costs. Although the combination of chemotherapy and phototherapy has the problem of light penetration depth, some medical equipment (such as gastroscope, endoscope, and optical fiber) can be used to transmit light to those deep lesions for the tumor that is located deep in the body. If some imaging functions can be integrated into such medical devices to track the position of phototherapy agents in real time, ensure that all tumor masses can be effectively exposed to light, and monitor the treatment response in real time, it will greatly promote the development of phototherapy in clinical cancer treatment.
(6) The metabolic pathways and phagocytosis mechanisms of some phototherapeutic agents in cells and in vivo have not been clarified. The photothermal-conversion efficiency and production of ROS by existing photothermal materials and PSs can still be improved, and new highly efficient phototherapeutic agents need to be developed. The visible light used in traditional PDT is not the ideal light source, and great efforts are still required to develop new generations of PDT agents that can be more effectively excited by the NIR light.

### 6.2. Further Perspectivesas 

A promising tumor combination treatment drug-delivery system, high drug-loading chemo–photo combination therapy nanomedicines need to be further developed by overcoming these problems.

(1) By understanding the combination therapy mechanism between chemotherapy and phototherapy in detail, the future development of chemo–photo combination therapy nanomedicines can give full play to the combination efficiency. Due to economy and convenience, the mathematical and computational modeling method has attracted more and more attention in the clinical translation of nanomedicine. It has been used to simulate nano-sized drug delivery to solid tumors in order to investigate efficacy, understand biological phenomena, and select optimal anticancer treatment strategies through different computational models, such as quantum mechanics, molecular dynamics, and monte carlo, which are related to pharmacokinetic/pharmacodynamics and so on [162,163]. For chemo–photo combination therapy, those nanomedicines can be examined and optimized through the integration of mathematical modeling techniques with modern imaging techniques and in vitro technologies to accelerate clinical translation of those nanomedicines.
(2) For the clinical application of chemo–photo combination therapy and the successful transition from laboratory to the clinic, further studying the safety and biocompatibility of nanomedicines and developing simple and inexpensive synthetic and highly repeatable methods to prepare photo combination therapy nanomedicines with higher efficiency combination therapy and lower side effects are urgent.
(3) The combination of imaging, tumor targeting, controlled drug release, and other functions of high drug-loading chemo–photo combination therapy nanomedicines are expected to further improve the tumor-treatment effect without significantly increasing the system complexity and reducing the drug-loading efficiency. Therefore, determining the optimum ratio between the carrier material and the drug is necessary to realize the multiple functions of simple structures. A novel type of high drug-loading nanomedicines that integrates diagnostic and therapeutic effects should be developed and manufactured, which can realize a real-time tumor diagnosis and treatment and achieve the best therapeutic effect in their institutes; drug carriers with “all in one” functions should be developed.
(4) The research of new therapeutic agents, especially photothermal and photodynamic agents, is still a very important research direction. Although there are very effective photothermal and photodynamic agents, they all have their own shortcomings, such as low solubility, low efficiency of photothermal and photodynamic treatment, and high dependence on tumor environment. The development of new photothermal and photodynamic agents can be greatly improved to enhance the effect of combined photothermal and photodynamic therapy.
(5) The development and application of multifunctional carrier-free nanomedicines (such as MOFs, π-π stacking and infinte coordation polymers) is an important research direction in this field because these kinds of nanomedicines have the advantages of a simple and green preparation method, high drug-loading efficiency, and few side effects. Technologies from different kinds of fields are required for these research directions. In this review, we hope researchers from various fields will join and collaborate on high drug-loading nanomedicine research for tumor chemo–photo combination therapy.

In summary, as the important part of chemo–photo combination therapy, high drug-loading chemo–photo combination therapy, high drug-loading nanomedicines have great advantages and potential in tumor therapy. Many innovative breakthroughs and discoveries will continue to be achieved through research on such nanomedicines in the future.

## Figures and Tables

**Table 1:** A summary of photothermal agents as carriers for high drug-loading nanomedicines.

Photoabsobers	Photothermal Agents	Chemotherapeutic Drug	Main Drug-Loading Mechanism	Drug-Loading Efficiency (wt%)	Ref.
Noble metal–based materials	AuNPs	Methotrexate	Electrostatic interactions	36.2%	[21]
	AuNRs	Doxorubicin	Electrostatic interactions	76.0%	[22]
	AuNBs	Doxorubicin	Electrostatic interactions	70.0%	[23]
	AuNFs	Doxorubicin	Electrostatic interactions	78.9%	[24]
	Au@Pt NPs	Doxorubicin	Electrostatic interactions	32.3%	[25]
	Pd@MnO_2_	Doxorubicin	Electrostatic interactions	58.0%	[26]
Transition metal–based materials	CuS NPs	Doxorubicin	Electrostatic interactions	55.5%	[27]
	MoS_2_	Doxorubicin	Electrostatic interactions	95.7%	[28]
	CoS, PDA	Doxorubicin	Electrostatic, π-π stacking	44.6%	[29]
	WS_2_ nanosheets	Doxorubicin	Electrostatic, π-π stacking	95.0%	[30]
	MoO_x_ nanosheets	Doxorubicin	Electrostatic, π-π stacking	65.0%	[31]
Carbon-based material	Nano-GO	Dacarbazine	π–π stacking	80.0%	[32]
	GDYO	Doxorubicin, cisplatin, methotrexate	Amide reaction, π-π stacking, electrostatic interactions	40.3% of Doxorubicin	[33]
	MCNs	Doxorubicin	Electrostatic, π-π stacking	69.2%	[34]
	CNTs	Doxorubicin	Electrostatic, π-π stacking	50.0%	[35]
	GQDs	Doxorubicin	Der Waals interaction, π-π stacking	96.6%	[36]
	SWNHs	Cisplatin and doxorubicin	Hydrophobic-hydrophobic, interactions and π-π stacking	52.4%	[37]
	mCNFs	5-Fluorouracil	electrostatic adsorption	31.0%	[38]
Organic nanomaterial	IR783	Camptothecin	Electrostatic, π-π stacking and hydrophobic interactions	62.0%	[39]
	ICG	Doxorubicin	Electrostatic, π-π stacking	58.2%	[40]
	IR1061	Paclitaxel	Electrostatic adsorption	59.3%	[41]
	PDA NPs	Doxorubicin	Coordinate bond, electrostatic adsorption	80.0%	[42]
	HMPAn NPs	Doxorubicin	Noncovalent electrostatic	37.5%	[43]
	PPY NPs	Doxorubicin	electrostatic adsorption	43.3%	[44]
Others	Iron oxide NPs	Curcumin	electrostatic adsorption	93.0%	[45]
	Ti-WC nanowires	Doxorubicin	π-π stacking	69.2%	[46]
	HM-Bi	Doxorubicin	electrostatic adsorption	78.0%	[47]

**Abbreviations:** AuNPs, gold nanoparticles; AuNRs, gold nanorods; AuNBs, gold nanobones, AuNFs, gold nanoframeworks; GO, graphene oxide; GDYO, graphdiyne oxide; MCN, mesoporous carbon nanospheres; CNTs, carbon nanotubes; GQDs, graphene quantum dots; SWNHs, single walled carbon nanohorns; mCNFs, mesoporous carbon nanoframes; HMPAn NPs, hollow mesoporous polyaniline nanoparticles; PPY, Poly(pyrrole-3-COOH); HM-Bi, hollow mesoporous bismuth nanoshells.

## Data Availability

Not applicable.
